# Specific Pharmacological Effects of Paroxetine Comprise Psychological but Not Somatic Symptoms of Depression

**DOI:** 10.1371/journal.pone.0159647

**Published:** 2016-07-20

**Authors:** Benjamin D. Schalet, Tony Z. Tang, Robert J. DeRubeis, Steven D. Hollon, Jay D. Amsterdam, Richard C. Shelton

**Affiliations:** 1 Department of Medical Social Sciences, Northwestern University Feinberg School of Medicine, Chicago, IL, United States of America; 2 Department of Psychology, University of Pennsylvania, Philadelphia, PA, United States of America; 3 Department of Psychology, Vanderbilt University, Nashville, TN, United States of America; 4 Depression Research Unit, Perelman School of Medicine, University of Pennsylvania, Philadelphia, PA, United States of America; 5 Department of Psychiatry and Behavioral Neurobiology, The University of Alabama at Birmingham, Birmingham, AL, United States of America; Radboud University Medical Centre, NETHERLANDS

## Abstract

**Background:**

Meta-analyses of placebo-controlled trials of SSRIs suggest that only a small portion of the observable change in depression may be attributed to "true" pharmacological effects. But depression is a multidimensional construct, so treatment effects may differ by symptom cluster. We tested the hypothesis that SSRIs uniquely alter psychological rather than somatic symptoms of depression and anxiety.

**Method:**

Outpatients with moderate to severe MDD were randomly assigned to receive paroxetine (*n* = 120) or placebo (*n* = 60).

**Results:**

Paroxetine significantly outperformed placebo on all psychological subscales of the syndrome measures, but not on any of the somatic subscales. The difference in score reduction between paroxetine and placebo was more than twice as great for the psychological symptoms compared to the somatic symptoms.

**Conclusions:**

Paroxetine appears to have a “true” pharmacological effect on the psychological but not on the somatic symptoms of depression and anxiety. Paroxetine's influence on somatic symptoms appears to be mostly duplicated by placebo.

## Introduction

Depression is a multi-faceted disorder encompassing emotional, cognitive, behavioral, and somatic symptoms. Treatment for major depression may include various forms of psychotherapy, antidepressant medication (ADM), such as the selective serotonin reuptake inhibitors (SSRIs), or a combination of both. Placebo-controlled clinical trials typically show that SSRIs and cognitive-behavioral therapies outperform placebo [1] [2].

One striking aspect of these clinical trials is the large symptom improvement in the placebo group. Meta-analyses of placebo-controlled trials of most SSRIs estimate that placebo accounts for about 75% of the effects of ADM during the acute phase treatment [3, 4]. That is, these data suggest that no more than 25% of the observable change may be attributed to the pharmacological effects of SSRIs, whereas the majority of change is due to nonspecific placebo effects and natural course of the illness (spontaneous remission). In this light, the psychopharmacological effects of SSRIs appear rather unimpressive.

This conclusion, however, is based exclusively on reported changes in total scores on depression outcome measures and treatment effects may differ by symptom clusters. The effectiveness of SSRIs for a wide range of mental disorders [5–8] indicates that they provide relief on diverse sets of psychological symptoms, or, alternatively, that they may alter broader dispositions, such as maladaptive personality traits [9–11]. Secondly, patients in depression studies rarely present exclusively with a “pure” set of depression symptoms, but nearly always have clinical or subclinical manifestations of other disorders, particularly anxiety [12], which may also be altered by SSRI treatment [13]. Finally, depression itself is a psychometrically multidimensional construct [14, 15] and improvement in one dimensional symptom set (such as mood) will not automatically accompany change in another (such as insomnia). To understand the scope and the limits of SSRI effects, researchers must examine outcomes in greater detail and depth.

In one such example, Tang et al. [9] examined both the depression severity and the personality trait of neuroticism in a placebo-controlled trial of paroxetine for moderate to severely depressed patients. Neuroticism refers to one’s tendency to experience exaggerated negative emotions of sadness, anger, and anxiety under conditions of stress [16, 17]. While 75% of the improvement observed with paroxetine on the traditional depression measure, the Hamilton Rating Scale for Depression (HRSD) [18, 19] was accounted for by placebo effect, only 23% of the observed decrease in neuroticism was duplicated in the placebo condition. In addition, the specific advantage for paroxetine over placebo with respect to depression was no longer significant after controlling for change in neuroticism, whereas its specific advantage over placebo in reducing neuroticism remained significant after controlling for change in depression.

It is possible that ADM substantially changes some depression symptoms and has virtually no effect (or a negative effect) on others. Consistent with this notion, meta-analysis of placebo-controlled SSRI trials show a wide range of effect sizes for the individual depression symptoms [20, 21]. For example, in two separate meta-analyses of ADM treatment studies of depression—one with tricyclics and the other with fluoxetine—Faries et al. [20] found that five symptoms (depressed mood, guilt, suicidality, disinterest / reduction in work and activities, and psychic anxiety) were more sensitive to differences between placebo and SSRIs compared to the other symptoms on the HRSD. Given that the HRSD is a commonly used measure in clinical trials [22], several researchers have promoted the use of different subscales of HRSD items on the basis of greater responsiveness to ADM, improved psychometric properties of these scales, and the association of individual items with overall depression severity [14, 20, 23–29].

While these studies generally demonstrate superior ADM effects for certain HRSD subscales, a clearly stated theory for the observed differential symptom effect sizes is still absent [29]. For example, in a post-hoc analysis, Fairies et al. [20] described the five symptoms common to the most responsive HRSD subscales as “core symptoms” of depression. This is equivalent to defining depression as that which SSRIs reduce, and implies that non-core symptoms are unimportant. A clear conceptual distinction is needed between those symptoms on which SSRIs have a “true” pharmacological effect versus those symptoms on which their effects are largely nonspecific.

We hypothesize that the specific pharmacological advantage of SSRIs over placebo will be largely concentrated on the *psychological* symptoms of depression and anxiety and not on the *somatic* symptoms. This counterintuitive hypothesis is consistent with an earlier finding that paroxetine has a considerably larger specific effect on neuroticism than on depression [9]. While four out of the nine depression symptoms articulated in the *DSM-IV* may be characterized as somatic [30], and somatic symptoms make up as many as 11 out of 17 symptoms assessed by the HRSD, they are entirely absent in neuroticism measurement. Somatic complaints have been found to correlate weakly with neuroticism, whereas psychological symptoms of both depression and anxiety correlate moderately to strongly [31, 32]. Finally, psychometric analyses indicate that somatic symptoms—fatigue, appetite loss/gain, insomnia, and anxious arousal—show relatively distinct patterns of association relative to the more general affective symptoms common to both depression and anxiety [14, 33–35].

We test this hypothesis on data generated in a placebo-controlled randomized trial of 180 moderately to severely depressed patients [2]. In addition, we will also explore how changes in the psychological subscales of depression and anxiety relate to changes in neuroticism, given their conceptual overlap [36].

## Method

### Participants

The Institutional Review Board at the University of Pennsylvania and the Human Research Protection Program (Institutional Review Board) at Vanderbilt University approved the study protocol and written informed consent was obtained from all participants. Subjects were moderate-to-severely depressed adult outpatients; patient characteristics, treatment procedure, and depression outcome findings have been detailed elsewhere [2, 37]. All patients met criteria for MDD and scored 20 or higher at both screening and intake evaluations on the 17-item version of the Hamilton Rating Scale of Depression [18, 19], modified to incorporate atypical symptoms [38]. Inclusion criteria were: (1) *DSM-IV* MDD diagnosis; (2) aged 18 to 70; (3) English speaking; and (4) willingness and ability to give informed consent. Exclusion criteria were: (1) history of bipolar I disorder; (2) substance abuse or dependence judged to require treatment; (3) current or past psychosis; (4) another *DSM-IV* Axis I disorder judged to require priority treatment; (5) antisocial, borderline, and/or schizotypal disorders (all other Axis II disorders were permitted); (6) suicide risk requiring immediate hospitalization; (7) a medical condition that contraindicated study medications; or (8) nonresponse to an adequate trial of paroxetine in the preceding year.

### Clinical Trial

The trial randomized 120 patients to paroxetine and 60 patients to pill-placebo. (Sixty patients were also randomized to a cognitive therapy group, but they are not included in this study of paroxetine mechanism). Thirteen paroxetine patients (11%) and eight placebo patients (13%) dropped out before week 8. The patients who dropped out did not differ significantly on depression or anxiety severity. Patients, psychiatrists, and evaluators were all blind as to whether the patients’ pills contained paroxetine. After week 8, the blind was broken and placebo patients were offered free medication treatment.

### Measurements

The following three symptom measures were administered both at intake and week 8: 17-item modified Hamilton Rating Scale for Depression (HRSD) [18, 19], modified to incorporate atypical symptoms [38], the 14-item Hamilton Rating Scale for Anxiety (HRSA) [39] and the 21-item Beck Anxiety Inventory (BAI) [40]. Both the HRSD and HRSA are clinician-administered measurements, while the BAI is self-reported. To maximize objectivity, clinicians who administered the HRSD and HRSA provided neither psychotherapy nor ADM treatment to participants in this study. Neuroticism was assessed at intake and week 8 using a 12-item scale from the NEO-Five-Factor Inventory (NEO-FFI) [41], a widely-used self-report measure based on the Five-Factor Model of Personality [42].

Among patients who continued with the study, some data were nonetheless missing, mostly anxiety scores at week 8. Because we intended to compare the magnitude of change across multiple measures and treatment conditions, we limited our analyses to patients who completed the HRSD, HRSA, and BAI at both intake and week 8. We excluded 7 placebo and 21 paroxetine patients from analysis (12% and 17% of intake, respectively), because these patients did not complete one or more questionnaires at either time point. Final sample sizes for analysis were 45 participants in placebo and 86 in paroxetine. The patients with missing questionnaire data did not differ significantly on intake depression or anxiety severity from the completer sample we analyzed.

For each of these three measures, we divided symptoms into somatic and psychological groups (Table 1) following the classification of Simon et al. [30] of the basic nine *DSM-IV* criteria for major depression. We excluded the HRSD hypochondriasis (#15) and insight (#17) items from our analyses, as they appeared unrelated to current *DSM-IV* criteria for major depression. Following the logic of Simon et al.’s [30] symptom division, we classified symptoms that primarily describe thoughts, moods, anxiety/fears, and interest/behaviour as psychological; symptoms that describe bodily manifestations were classified as somatic (e.g., fatigue, hypersomnia, changes in weight, heart racing).

**Table 1:** Classification of Depression and Anxiety Symptoms as Psychological or Somatic, by Instrument Item Numbers.

Symptom	HRSD Item #	HRSA Item #	BAI Item #
Psychological			
Anxiety Psychic	10	1	10
Concentration		5	
Depressed Mood	1	6	
Fears		3	5,9,14,16,17
Guilt	2		
Suicide (thoughts and behavior)	3		
Work & Activities (loss of interest)	7		
Somatic			
Agitation / Tension	9	2	4
Anxiety Somatic	11	7,8	
Appetite Decrease/Increase	12,12A		
Autonomic (e.g., dry mouth)		13	20,21
Behavior at Interview (e.g., fidgeting, hand tremor)		14	
Cardiovascular (e.g., heart racing)		9	7,19
Dizzy/Lightheaded			6
Fatigue/Energy Loss	13		
Feeling Hot			2
Gastrointestinal (e.g., abdominal pain)		11	18
Genitourinary (e.g., frequent urination)		12	
Hands Trembling			12
Hypersomnia	4A-6A		
Insomnia	4–6	4	
Libido / Interest in Sex	14		
Numbness / Tingling			1
Respiratory (e.g., choking feeling)		10	11,15
Retardation	8		
Shaky / Unsteady / Wobbliness			3,8,13
Weight Gain/Loss	16,16A		
Residual			
Hypochondriasis	15		
Insight	17		

Numbers represent the item number of the symptom in each scale. Abbreviations: BAI, Beck Anxiety Inventory; HRSA, Hamilton Rating Scale of Anxiety; HRSD, 17-item version of the Hamilton Rating Scale for Depression, modified to incorporate atypical symptoms.

**Fig 1:**
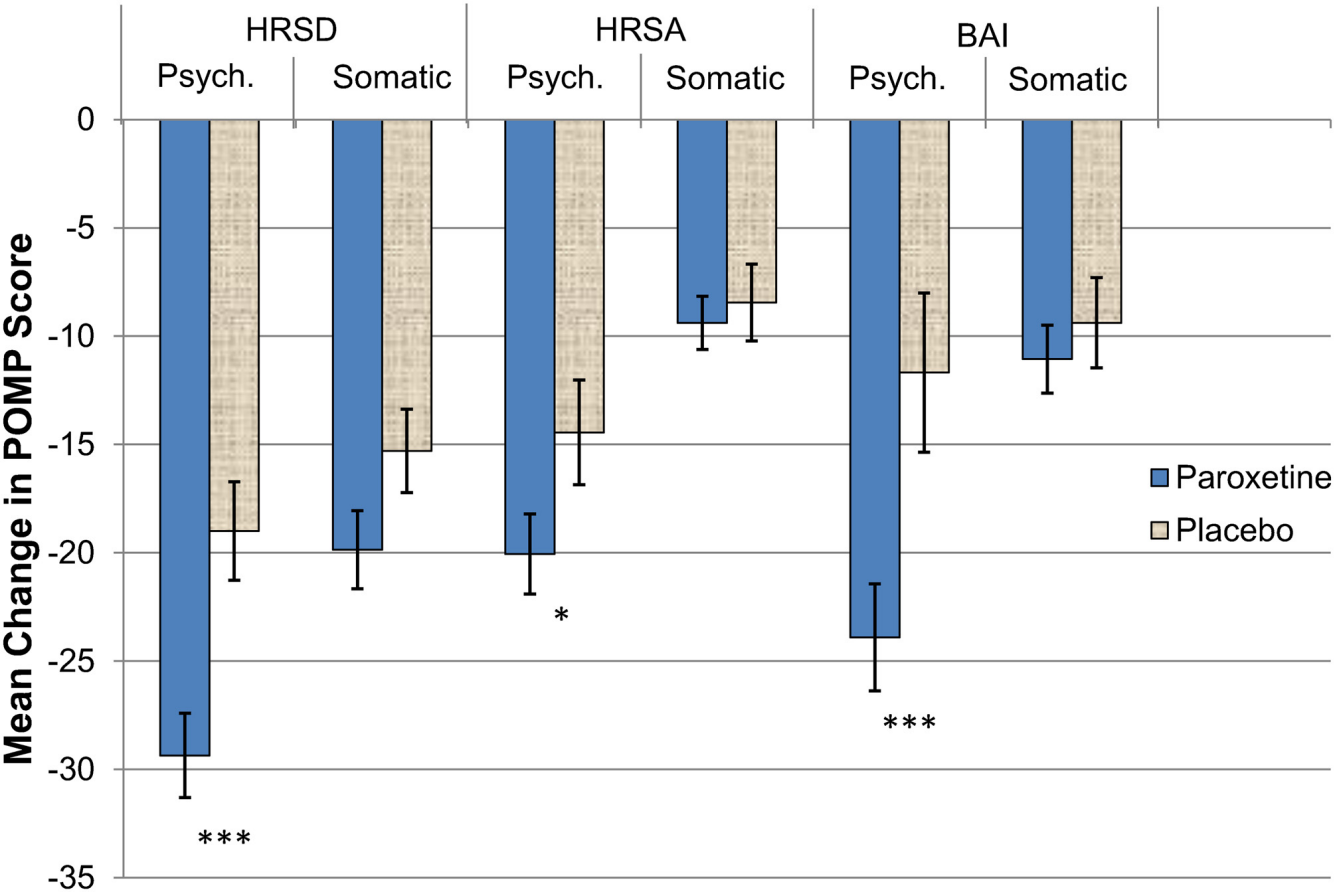
Reduction in percentage of maximum possible (POMP) score from intake to week 8 in placebo and paroxetine conditions. Error bars represent standard errors of the mean difference score. Abbreviations: BAI, Beck Anxiety Inventory; HRSA, Hamilton Rating Scale of Anxiety; HRSD, 17-item version of the Hamilton Rating Scale for Depression, modified to incorporate atypical symptoms; psych., psychological. * *p* <.05 paroxetine vs. placebo; *** *p* <.001 paroxetine vs. placebo.

Both libido (HRSD #14) and "unable to relax" (BAI #4) were classified as somatic; however, we recognize that these classifications might be challenged. Although we classified the HRSD libido item as somatic (as did Enns et al. [31]), the HRSD interview emphasizes interest in and thoughts about sex, not sexual performance. The BAI item "unable to relax" could possibly refer to cognitive manifestations rather than bodily tension. Nevertheless, placing these two items in the psychological subscales does not change the results; indeed, these items show changes that lie somewhere between the average changes we report for their respective somatic and psychological subscales (Fig 1).

Our modified HRSD [38] included the assessment of three atypical symptoms of depression: hypersomnia, weight gain, and appetite increase, along with their typical counterparts (insomnia, weight loss, and appetite decrease). Following Reimherr et al. [38] and DeRubeis et al. [2], only the maximum of each patient's typical/atypical pair (e.g., weight gain or loss) was added into the total of both the HRSD full scale and the somatic subscale. We considered other scoring options, but this scoring method produced the result that was *least* favorable to our hypothesis. For example, if only the typical or the atypical symptoms are included in the subscale, the non-significant medication advantage over placebo (Fig 1) is further cut in half.

### Descriptive Analysis

Typically, effect sizes in treatment studies are calculated by dividing the difference of two group means by the pooled standard deviation (SD) [43]. Unfortunately, standardized effects also pose several problems, especially when the purpose is to compare effects across different measures, samples, or conditions. Effect sizes may be sensitive to particular sample characteristics (such as restricted range) and differences in the reliability of the measures [44]. In addition, standardizing essentially erases scale anchors (e.g., "not at all", "moderately", "severely"), which are still meaningful in average item scores. Reporting change in average symptom scores, however, is complicated by the fact that the rating scales of each symptom differ across the three measures and, in the case of the HRSD, also within the measure. Although HRSD total scores have established ranges corresponding to depression severity [45], these ranges have not been established for somatic and psychological subscales.

To place scores and changes therein on comparable units, we converted scores on the subscales of the HRSD, HRSA, and BAI to Percent of Maximum Possible (POMP) scores [46, 47]. POMP scores are calculated by linearly transforming each participant’s raw score into a percentage of the maximum possible total score of the measure. Descriptive statistics of POMP scores are informative with respect to the range of all possible scores (and the implied severity), and readily comparable to POMP scores of other samples and similar measures [46, 47]. POMP scores do not alter inferential statistics like t- or F-tests and are increasingly applied in clinical research [17, 48–51]. To facilitate any possible direct comparison with other studies, we reported POMP scores separately for subscales of each measure, rather than combining similar subscales or items across measures.

### Inferential Analysis

To test for significant differences among treatment conditions, we completed standard ANCOVA, with treatment condition as the independent variable, the symptom measures at week 8 as dependent variables, and intake symptom measures as a covariate. We completed these tests separately for the HRSD, HRSA, and BAI total scores, and the respective psychological and somatic subscales. Expecting significant differences for the psychological measures as dependent variables, we added neuroticism intake and week 8 scores as covariates. We also completed the reverse analysis to see if treatment assignment would still predict neuroticism reduction (relative to placebo) after controlling for psychological symptom improvement. Finally, we calculated Cohen’s d for placebo vs. active treatments by dividing the difference in the respective least squares means at week 8 by the pooled SD of the respective means. Such effects can be considered large when they are 0.8 and above, medium between 0.5 and 0.8, and small between 0.2 and 0.5 [43].

## Results

### Main Effects

Fig 1 illustrates the discrepancy in the unique effect of paroxetine on psychological compared to somatic symptoms during the acute phase of therapy. (Table 2 also shows intake and week 8 means and standard deviations for the total scale measures and their somatic and psychological subscales). For depression, the difference in score reductions between paroxetine and placebo was more than twice as great for the psychological symptoms as for the somatic symptoms: 10.4% vs. 4.6% (of maximum possible scores). The contrast between the advantage for paroxetine in psychological symptoms vs. somatic symptoms was even stronger for the anxiety scales: 12.2% vs. 1.7% on the BAI and 5.6% vs. 0.9% on the HRSA.

**Table 2:** Intake and Week 8 Scores on Symptom Measures, as Percentage of Possible Maximum (POMP) Scores.

	Intake		Week 8	
Total Scale	Placebo	Paroxetine	Placebo	Paroxetine
HRSD				
Mean	44.6	44.5	29.5	22.8
SD	4.5	5.4	12.2	12.5
HRSA				
Mean	30.8	28.8	20.7	16.3
SD	10.1	9.3	11.5	10.0
BAI				
Mean	23.7	24.2	13.6	9.4
SD	13.8	15.4	13.8	10.1
Psychological Subscales				
HRSD				
Mean	54.3	51.7	35.3	22.3
SD	9.3	9.2	16.4	16.6
HRSA				
Mean	40.7	39.3	26.3	19.3
SD	11.4	10.7	15.1	14.6
BAI				
Mean	33.0	33.5	21.4	9.6
SD	24.6	22.3	24.0	13.1
Somatic Subscales				
HRSD				
Mean	45.8	47.5	30.5	27.6
SD	7.0	9.0	13.3	14.5
HRSA				
Mean	26.9	24.6	18.4	15.2
SD	10.9	10.8	11.6	10.0
BAI				
Mean	19.9	20.4	10.6	9.4
SD	12.5	15.6	11.3	10.5

Abbreviations: BAI, Beck Anxiety Inventory; HRSA, Hamilton Rating Scale of Anxiety; HRSD, 17-item version of the Hamilton Rating Scale for Depression, modified to incorporate atypical symptoms.

Table 3 shows the main effect statistics for the treatments on the symptom scales. F-tests on all symptom measures indicated that paroxetine significantly outperformed placebo on total HRSD scores (*p* = .004), BAI scores (*p* = .03), and marginally on total HRSA scores (*p* = .054). Paroxetine also significantly outperformed placebo on all psychological subscales, (*p* <.001 for HRSD, *p* = .01 for the HRSA, *p* <.001 for the BAI), but it did not show significant advantage over placebo on any of the somatic subscales (*p*s ≥.20). In short, paroxetine outperformed placebo substantially on the psychological subscales, but it did not outperform placebo on the somatic subscales.

**Table 3:** Paroxetine vs. Placebo Main Effects for Depression, Anxiety, and Subscales.

	Paroxetine vs. Placebo		
Dependent Variable	F	P	ES
Total measures			
HRSD	8.7	0.004	0.54
HRSA	3.8	0.05	0.36
BAI	4.8	0.03	0.4
Psychological Subscales			
HRSD-psych.	15.9	<.001	0.74
HRSA-psych.	6.2	0.01	0.46
BAI-psych.	15.3	<.001	0.72
Somatic Subscales			
HRSD-somatic	1.5	0.22	0.23
HRSA-somatic	1.7	0.2	0.24
BAI-somatic	0.5	0.47	0.13

Abbreviations: BAI, Beck Anxiety Inventory; HRSA, Hamilton Rating Scale of Anxiety; 17-item version of the Hamilton Rating Scale for Depression, modified to incorporate atypical symptoms; psych., psychological.

### Change in Psychological Symptoms vs. Neuroticism

Consistent with previous research [31, 32], we found that neuroticism correlated more closely with our psychological subscales than with somatic subscales, both at intake (Mean *r* = .32 vs. Mean *r* = .07) and at week 8 (Mean *r* = .45 vs. Mean *r* = .29), with all differences between corresponding psychological and somatic correlations being significant, all *p*s <.05.

In an effort to understand the level of overlap between neuroticism and the psychological symptom scales, we repeated our ANCOVA of treatment assignment (paroxetine vs. placebo), but with additional covariates. First, we found that treatment assignment (paroxetine vs. placebo) had a unique effect on neuroticism reduction, even after controlling for the psychological symptoms (*p*s ≤.02). (See Table 4 for details). Even when all three psychological symptom measures are entered simultaneously as covariates, paroxetine still significantly outperformed placebo in reducing neuroticism (*p* = .01) suggesting that paroxetine’s effect on neuroticism is not a mere byproduct of psychological symptom change. On the other hand, for the psychological symptoms of HRSD and BAI, the differences between reduction on paroxetine and placebo could not be entirely explained by neuroticism reduction either (*p* = .03 and *p* = .02, respectively). However, when controlling for neuroticism, treatment assignment no longer predicted reduction on the HRSA (*p* = .65).

**Table 4:** Paroxetine vs. Placebo Comparison of Symptom Measure and Neuroticism Change.

Dependent Variable	Covariate^a^	*F*	*P*	ES
Personality				
Neuroticism	HRSD-psych.	5.92	0.02	0.5
Neuroticism	HRSA-psych.	12.48	<.001	0.68
Neuroticism	BAI-psych.	9.66	0.002	0.62
Neuroticism	All 3 psych scales	6.4	0.01	0.53
Subscales				
HRSD-psych.	Neuroticsm	4.66	0.03	0.44
HRSA-psych.	Neuroticsm	0.24	0.65	0.1
BAI-psych.	Neuroticsm	5.2	0.02	0.46

Abbreviations: BAI, Beck Anxiety Inventory; HRSA, Hamilton Rating Scale of Anxiety; HRSD, 17-item version of the Hamilton Rating Scale for Depression, modified to incorporate atypical symptoms; psych., psychological.

^a^For each comparison, covariates consist of the dependent variable intake score, as well as both the intake and week 8 scores of scales listed in the covariate column.

## Discussion

In this study, we analyzed symptom improvements in a moderately-to-severely depressed sample during placebo and paroxetine treatments. To our best knowledge, this study is the first to characterize the SSRI advantage over placebo as primarily psychological rather than somatic. In fact, differences in treatment assignment were not significant for any of the somatic subscales. In addition, differences between patients in the paroxetine condition and the placebo condition were much greater on psychological symptoms than on somatic symptoms, particularly for anxiety (Fig 1).

Our results are consistent with past research that empirically searched for HRSD items showing the largest treatment effects, typically without considering the nature of the HRSD items [20, 27]. For example, the top five items identified by Faries et al. [20] and Entsuah et al. [27], as well as five of the top six items identified by Santor et al. [29] belong to our psychological subscales of the HRSD. Our results extend these results beyond the HRSD to the HRSA and BAI. More importantly, it now gives a possible theoretical rationale to these past findings.

Fournier et al. [35] also analyzed the HRSD items of this clinical trial. Unlike this study, however, Fournier et al. [35] divided the 24-item version of the HRSD symptoms into five clusters using factor analysis. These empirically derived clusters show a somewhat complex relationship with the system of psychological vs. physiological items in this project. For example, their 3-item mood cluster includes depressed mood, anhedonia, and loss of energy. In this project, depressed mood and anhedonia are classified as psychological, but loss of energy is classified as a somatic symptom, consistent with the symptom divisions of Simon et al. [30], Shafer [15], and Enns et al. [31]. Fournier et al.’s [35] five-item cognitive/suicide cluster included only items from our psychological symptom subscale: suicide and guilt, along with hopelessness, helplessness, and worthlessness from the 24-item HRSD. Consistent with our results and theory, this cluster showed the largest effect size in favor of paroxetine over placebo among the five clusters at week 8; and changes in the other clusters (all of which included one or more somatic symptoms) did not differ significantly from placebo.

### Separate Neurobiological Correlates

Our findings indicate that SSRI treatment differentially impacts psychological and somatic symptoms of depression and anxiety, showing much greater specific effects (relative to placebo) on psychological symptoms. One of the potential mechanisms for this finding is that separate neurobiological structures and pathways may be implicated in the expression of psychological versus somatic symptoms. Thus, while depressed mood may be marked by abnormal activation of the medial prefrontal cortex and difficulty concentrating is strongly associated with hypoactivity in the dorsolateral prefrontal cortex [52], motor retardation may be embodied by dysregulation in the striatum and physical tiredness may be associated with dopamine depletion in nucleus accumbens [53].

Antidepressant medications have been thought to act predominantly on neurovegetative symptoms of depression [54]. However, these effects are primarily associated with the older tricyclic antidepressants. Tricyclics block the reuptake of norepinephrine and serotonin, but more predominantly act on norepinephrine. Further, tricyclics are potent antagonists of histamine-1 receptors, conferring strong sedating properties. By contrast, SSRIs like paroxetine have little if any actions on either norepinephrine reuptake or histamine receptors [55]. Paroxetine is the most potent inhibitor of the norepinephrine transporter of all SSRIs but the actions on serotonin are 10-fold greater on than norepinephrine [55].

Why should SSRIs act preferentially on psychological symptoms of depression? In 1986 Depue and Spoont proposed that serotonin has an effect of constraining both behavioral inhibition and behavioral facilitation systems [56]. This concept was supported subsequently by Knutson et al. [57], who showed a general reduction in negative affect with paroxetine, and by Sheline et al. [58] who showed that the SSRI sertraline inhibited the excess left amygdala response to all faces, particularly fearful faces using fMRI. These effects are also consistent with the observations of Tang et al. of the effects of paroxetine on neuroticism noted above. This inhibiting effect of serotonin on amygdala reactivity and general distress symptoms is attributable to the actions on specific serotonin receptors on inhibitory, GABAergic interneurons [59]. Serotonin has a complex role in CNS function given the large number of serotonin receptors in brain and their sometimes opposing roles [60]. However, the current work continues to support the original Depue and Spoont [56] notion of an overall constraining effect on distress-inducing brain regional activity.

### Treatment Implications

Our results suggest that the effects of SSRIs on somatic symptoms are not stronger than that of placebo. Researchers and clinicians may need to look towards additional medications to reduce these symptoms further. While neurological evidence for distinct systems is still limited, it stands to reason that improvement on somatic symptoms (such as fatigue) may require separate treatments from those that address psychological symptoms. One approach may be to target multiple neurotransmitter pathways; duel-acting medications (such as duloxetine and venlafaxine) may be more effective than SSRIs in treating some somatic symptoms of depression [61–64], though the advantage over SSRIs in total depression scores is rather modest [65].

Research on ADM treatment of low energy levels specifically in the context of depression has been limited, despite its apparent centrality to major depression. Not surprisingly, low energy (or fatigue) is among the most common residual symptom after acute SSRI treatment [66]. Buproprion, a norepinephrine and dopamine reuptake inhibitor that targets the frontal cortex may be more effective in improving energy levels than standard SSRIs [67, 68]. A meta-analysis of duloxetine trials shows a moderate improvement on the somatic HRSD symptoms of energy and retardation, though this holds primarily for moderate to severely depressed patients [64]. In addition, first-line ADM treatment may be augmented with modafinil or central nervous system stimulants, which promote wakefulness [66, 69].

### The Role of Neuroticism

Our results are consistent with earlier findings that SSRIs may directly target neuroticism, a broad disposition to experience negative emotions [9, 10] that includes no somatic content [16, 41]. Our psychological subscales, therefore, show more efficient empirical and conceptual overlap with neuroticism compared to the full-length symptom scales. Although measures of neuroticism, depression, and anxiety exhibit considerable construct overlap, neuroticism (as a personality measure) is nevertheless crucially different from symptom measures because of the absence of any time-frame context [36]. A future area of investigation would be to further understand the extent to which treatment effects may be attributed to such unique aspects of personality assessment or to the intersection of personality and depression/anxiety symptoms.

### Limitations

Our study needs to be replicated using both paroxetine and other SSRIs to determine if the effects are reliable and if they are limited to a single medication or medication class [70]. The use of multiple and more comprehensive measures of depression and anxiety would also have increased confidence in the finding. In addition, we did not directly measure the neurobiological systems underlying psychological and somatic symptoms. We also did not have plasma levels of paroxetine available to verify compliance. It is arguable that splitting each instrument into two subscales increased the type-1 error rate. However, setting alpha at 0.025 (instead of 0.05) for significance testing alters the conclusion of only one test, namely, that paroxetine no longer predicts change in psychological symptoms of the HRSD when controlling for Neuroticism change. Finally, there were a number of dropouts in the trial (11–13%) and our samples were modest and limited to those who completed assessments at both time points.

### Significance

Although SSRIs are the most widely prescribed treatment for major depression, the field still needs a comprehensive description and clearly stated formulation of the symptom changes caused by SSRIs. Our results contribute to this effort by suggesting that SSRIs enact improvement beyond placebo mostly for psychological, but not somatic symptoms of depression and anxiety. To further improve somatic symptoms of depression, researchers may need to further explore how to improve treatments of somatic symptoms.

## Supporting Information

S1 Data SPSS data file containing variables used in analysis (deidentified data). (SAV)

S1 Data Syntax Text file containing SPSS commands used in analysis. (TXT)
